# An open-label, multicenter evaluation of the long-term safety and efficacy of risperidone in adolescents with schizophrenia

**DOI:** 10.1186/1753-2000-6-23

**Published:** 2012-06-07

**Authors:** Gahan Pandina, Stuart Kushner, Keith Karcher, Magali Haas

**Affiliations:** 1 Janssen Research and Development, LLC, 1125 Trenton-Harbourton Road, Titusville, NJ, 08560, USA

**Keywords:** Adolescent schizophrenia, Risperidone, Long-term treatment

## Abstract

**Background:**

Data on the long-term efficacy, safety, and tolerability of risperidone in adolescents with schizophrenia are limited. The objective of this study was to evaluate the efficacy and safety of maintenance risperidone treatment in adolescents with schizophrenia.

**Methods:**

This open-label study of adolescents aged 13 to 17 years with schizophrenia was a single extension study of two short-term double-blind risperidone studies and also enrolled subjects directly in open-label risperidone treatment. The risperidone dose was flexible and ranged from 2 to 6 mg/day. Most subjects enrolled for 6 months; a subset enrolled for 12 months. Assessment tools included the Positive and Negative Syndrome Scale total and factor scores, Clinical Global Impressions, Children’s Global Assessment Scale, adverse event (AE) monitoring, vital signs, laboratory testing, and extrapyramidal symptom rating scales.

**Results:**

A total of 390 subjects were enrolled; 48 subjects had received placebo in a previous double-blind study; 292 subjects had received risperidone as part of their participation in one of two previous controlled studies; and 50 subjects were enrolled directly for this study. A total of 279 subjects enrolled for 6 months of treatment, and 111 subjects enrolled for 12 months of treatment. Overall, 264 (67.7%) subjects completed this study: 209 of the 279 subjects (75%) in the 6-month group and 55 of the 111 subjects (50%) in the 12-month group. The median mode dose was 3.8 mg/day. At 6 months, all three groups experienced improvement from open-label baseline in symptoms of schizophrenia as well as general assessments of global functioning. Improvements were generally maintained for the duration of treatment. The most common AEs (≥10% of subjects) were somnolence, headache, weight increase, hypertonia, insomnia, tremor, and psychosis. Potentially prolactin-related AEs (PPAEs) were reported by 36 (9%) subjects. The AE profile in this study was qualitatively similar to those of other studies in adult subjects with schizophrenia and in other psychiatric studies of risperidone in pediatric populations.

**Conclusions:**

Risperidone maintenance treatment in adolescents over 6 to 12 months was well tolerated, consistent with related studies in this clinical population, and associated with continued efficacy.

**Clinical trials:**

ClinicalTrials.gov registration number: NCT00246285 http://clinicaltrials.gov/ct2/show/NCT00246285?term=NCT00246285&rank=1

## Background

Schizophrenia is a complex and severe neurodevelopmental brain disorder that generally has a chronic course resulting in significant long-term morbidity and functional impairment. Onset of symptoms is most common in late adolescence or early adulthood [1], although only an estimated one in 10,000 children worldwide meet full criteria for a formal diagnosis of schizophrenia [2,3] with an increase in frequency between 13 and 18 years of age [2]. Child or adolescent onset is usually associated with longer treatment delays than adult onset [4].

Antipsychotic medication is generally accepted as a critical piece of a comprehensive care approach for younger populations with schizophrenia [5-11]. Long-term safety and tolerability in pediatric patients with schizophrenia is of paramount concern for clinicians, given that long-term antipsychotic treatment is the standard of care. Younger populations may be more susceptible than adults to treatment-related adverse events (AEs) [12-14]. AEs of particular interest include extrapyramidal symptoms (EPS), somnolence/fatigue, weight gain, effects on glucose and lipid metabolism, prolactin elevation and potentially prolactin-related AEs, and the potential for effects on growth and sexual maturation. Although several studies have previously documented the safety and tolerability of risperidone in disruptive behavior disorders over a period of 1 year or longer [15,16], data on the long-term safety and tolerability of risperidone in adolescents with schizophrenia are more limited.

Two randomized, double-blind, controlled studies have demonstrated the short-term efficacy and safety of risperidone in adolescents with schizophrenia [8,9]. Doses evaluated were similar to those used typically in the treatment of adults, ranging from 1 to 6 mg/day.

The aim of this open-label study of risperidone in adolescents with schizophrenia, which included a subgroup treated for up to 12 months, was to examine whether adolescent patients experienced continued benefits of risperidone treatment without the emergence of new or unexpected safety issues.

## Methods

### Study design

This open-label, multicenter study (NCT00246285) was conducted in 12 countries (Belgium, Bulgaria, Czech Republic, Estonia, Germany, India, Poland, Romania, Russia, Spain, Ukraine, and the United States) from May 29, 2001, to December 20, 2006. The study protocol and amendments were approved by institutional review boards or independent ethics committees before study initiation, and the study was conducted in accordance with the Declaration of Helsinki. All subjects gave consent to participate; their legal representatives provided written informed consent before any study procedures were initiated.

Changes to the study design were made to fulfill regulatory requirements. The changes consisted of a reduction in the planned duration of treatment and the dose range. The planned duration of treatment was initially 12 months but was subsequently changed to 6 months in a protocol amendment (amendment 3). Subjects who were enrolled before the amendment were treated for up to 12 months. Amendment 3 also changed the maximum dose from 4 to 6 mg/day and the dose range from 0.03 to 0.08 mg/kg/day to 2 to 6 mg/day.

### Subjects

**Figure 1:**
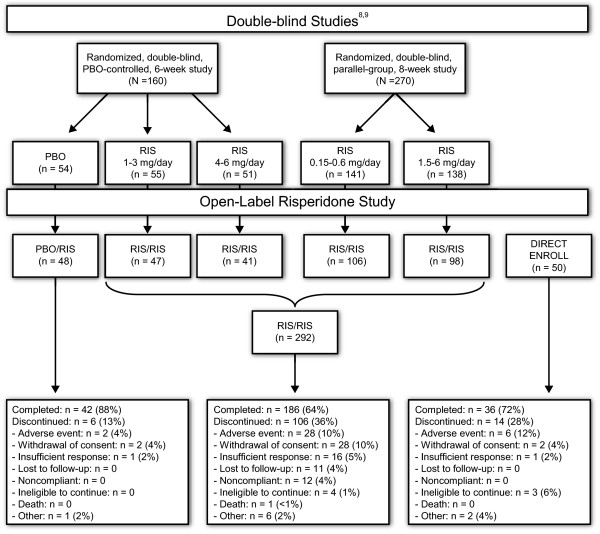
Study scheme and overall disposition

Several sources of enrollment into the trial were possible. Adolescents with schizophrenia were enrolled directly into the open-label study; in addition, subjects were enrolled after completing their participation in one of two short-term, double-blind, controlled clinical studies (NCT00088075 and NCT00034749) assessing the short-term efficacy and safety of risperidone (Figure 1) [8,9]. 

Inclusion and exclusion criteria for the previous double-blind studies have been described in detail [8,9]. Subjects in the two double-blind studies were eligible for the open-label study if they had completed at least 24 days of previous double-blind treatment or discontinued because of tolerability issues, if they were expected to benefit from continuation of treatment, and if they had no other serious, unstable illnesses and were otherwise physically healthy.

Additional subjects were enrolled directly into the open-label study. Inclusion and exclusion for directly enrolled subjects were similar to those of the double-blind studies. Subjects were aged 13 to 17 years, of either sex, and in good physical health with no serious illnesses or neurologic conditions other than schizophrenia. Subjects were diagnosed with schizophrenia (according to *Diagnostic and Statistical Manual of Mental Disorders*, 4th edition *DSM-IV* criteria) using the semistructured clinical interview for *DSM-IV* for children of the Kiddie Schedule for Affective Disorders and Schizophrenia, Present and Lifetime version (K-SADS-PL). Training on the proper use of the K-SADS-PL was provided during investigator meetings and via Internet-based training sessions. Reliability was determined by independent review by an expert panel of the first K-SADS-PL completed after training. Additionally, subjects had to have a Positive and Negative Syndrome Scale (PANSS) [17] total score of 40 to 120 at screening and baseline. Subjects already receiving oral risperidone could be enrolled only if they were expected to benefit from continued treatment. Exclusion criteria included diagnosis of dissociative disorder, bipolar disorder, major depressive disorder, schizoaffective disorder, schizophreniform disorder, autistic disorder, or primary substance-induced psychotic disorder. Subjects were also excluded if they had mental retardation (intelligence quotient <70), if they had known or suspected substance dependence, or if they were considered at significant risk of suicide or violent behavior. Subjects were ineligible if more than 1 week had elapsed since completing or discontinuing from the short-term study.

### Study treatment

Directly enrolled subjects and those who had taken study medication in tablet form during the 6-week, double-blind, placebo-controlled study [9] received an initial risperidone dose of 0.5 mg in tablet form on day 1 of the OL phase. Subjects who had taken an oral risperidone solution in the 8-week double-blind study [8] received an initial risperidone dose of 0.01 mL/kg in liquid form on day 1 of the OL phase.

By day 7, risperidone was titrated for all subjects to a minimum of 2 mg/day, followed by titration to a maximum tolerated dose between 2 and 6 mg/day. Dosage was under the control of each site’s investigators, who adjusted each subject’s dosage on the basis of their assessment of the efficacy and tolerability of the study medication. Once a stable dose was achieved, this dose was maintained for the rest of the study and adjusted only in the case of emergent tolerability or efficacy issues. Subjects who could not be maintained on a minimum of 2 mg/day were withdrawn from the study.

Except for other antipsychotics, the concomitant use of other psychotropic treatments, such as antidepressants, mood stabilizers, and anxiolytics, were allowed. These medications and respective dosing regimens were provided to patients per the local regulatory guidelines of the individual countries and per clinician judgment. The flexibility in concomitant therapy allows investigators to treat each individual to a level that is optimal for that subject.

### Study assessments

Efficacy parameters assessed were PANSS total and factor scores for positive symptoms, negative symptoms, disorganized thoughts, uncontrolled hostility/excitement, and anxiety/depression [18]; clinical response (defined as ≥20% reduction from open-label baseline in mean PANSS total score), Clinical Global Impressions for Severity and Clinical Global Impressions for Improvement [19]; and the Children’s Global Assessment Scale (CGAS) [20]. Investigators received training and certification to ensure consistent administration and scoring of the PANSS. Certification of raters consisted of detailed instruction on the PANSS, including a focus on developmental adjustments. Following training, raters were tested by scoring a tape of adolescents with schizophrenia who demonstrated sufficient positive and negative symptoms. Rater candidates were certified by adequate scoring of this interview and by demonstrating appropriate credentials and experience. Retraining occurred at least annually. To the extent possible, assessments were made at approximately the same time of day and by the same clinician at all visits.

Safety and tolerability assessments included AE monitoring, laboratory tests, vital signs, body weight and height, physical examination, Tanner staging [21,22], and electrocardiograms. EPS severity was assessed by the Abnormal Involuntary Movement Scale (AIMS) [23], the Simpson Angus Scale (SAS) [24], and the Barnes Akathisia Rating Scale (BARS) [25]. The sexual maturity of subjects was assessed by a qualified physician using Tanner staging which rates on a scale of 1 to 5 through the selection of one diagram (from a series of five) thought to most closely resemble the sexual maturity of the subject. AEs of potential clinical interest (including somnolence, fatigue, EPS-related AEs, potentially prolactin-related AEs, and glucose metabolism–related AEs) were grouped together in categories by the World Health Organization Adverse Reaction Terminology–preferred terms and examined separately.

### Data analysis

Efficacy and safety were analyzed in the intent-to-treat population (all subjects who received at least one dose of risperidone).

For all subjects who entered the open-label study from a previous double-blind trial, the final efficacy assessments in the double-blind study served as the baseline assessments for the open-label study. All efficacy analyses included changes from open-label baseline based on both observed and last-observation-carried-forward values. The month 6 end point (defined as the last nonmissing, postbaseline value that fell on or before month 6) and the overall end point (the last nonmissing, post–open-label baseline value) were included in all efficacy summaries. Month 12 results for subjects who were enrolled before the protocol was amended were also summarized. Results are summarized for all subjects and for three subject groups: subjects previously randomly assigned to receive placebo (the PBO/RIS group), subjects previously randomly assigned to receive risperidone (the RIS/RIS group), and directly enrolled subjects (the direct-enroll group). Change from baseline within subject groups and for all subjects was analyzed using paired *t* tests. Because several sources of trial enrollment were possible and because subjects were not randomly allocated to the different treatment groups, no between-subject group statistical comparisons were made.

For somnolence AEs, time to first event was assessed graphically using Kaplan-Meier curves. Weight and body mass index (BMI) were transformed to *z* scores based on the United States 2000 Centers for Disease Control and Prevention growth charts (http://www.cdc.gov/growthcharts). The *z* score indicates how many standard deviations (SDs) an observed value is away from the expected weight or BMI, based on a subject’s age (in months) and sex: no deviation from expected weight or BMI results in a *z* score change of 0 (SD = 1).

## Results

### Subjects and disposition

The intent-to-treat population consisted of 390 subjects (Figure 1). A total of 50 were directly enrolled (direct-enroll group); 136 entered from the double-blind, placebo-controlled, 6-week study (including 48 who had received placebo [PBO/RIS group] and 88 who had received risperidone) [9]; and 204 entered from the double-blind, 8-week study (all of whom had received risperidone, some at doses as low as 0.15 to 0.6 mg/day) [8]. The RIS/RIS group comprised the 292 subjects who had previously received risperidone. A total of 111 subjects in the RIS/RIS group enrolled for 12 months of treatment before the protocol change. These subjects had all been enrolled in the 8-week study.

Overall, 264 (67.7%) subjects completed the study per protocol (either 6 or 12 months); 42 subjects (88%) PBO/RIS; 186 subjects (64%) RIS/RIS; and 36 subjects (72%) direct-enroll. A total of 126 subjects discontinued the study: 6 subjects (13%) PBO/RIS, 106 subjects (36%) RIS/RIS, and 14 subjects (28%) direct-enroll. AEs were the most frequent reason for discontinuation in all groups (Figure 1). Of the 111 subjects enrolled for 12 months before the protocol amendment, 55 (50%) completed 12 months of treatment compared with 209 (75%) who enrolled after the protocol amendment and completed 6 months of treatment. The most common reason for discontinuation for the 12-month group was AEs (18 subjects [16.0%]).

Table 1 summarizes demographic parameters and baseline characteristics. Mean age was 15.5 years, and the majority of subjects were male (61%). The mean age at onset of first psychotic symptoms was 13.3 years, average age at diagnosis was 14.9 years, and mean age at the start of antipsychotic treatment was 14.5 years.

**Table 1:** Baseline demographic and clinical characteristics of adolescents with schizophrenia

**Parameter**	**PBO/RIS**	**RIS/RIS**	**Direct-enroll**	**All subjects**
	**(n = 48)**	**(n = 292)**	**(n = 50)**	**(N = 390)**
Age, mean (SD), y	15.4 (1.4)	15.5 (1.7)	15.5 (1.4)	15.5 (1.6)
Sex, n (%)				
Female	18 (38)	114 (39)	20 (40)	152 (39)
Male	30 (63)	178 (61)	30 (60)	238 (61)
Weight, mean (SD), kg	59.2 (20.9)	60.3 (13.4)	67.3 (14.2)	61.0 (14.8)
Body mass index, mean (SD), kg/m^2^	21.8 (5.9)	21.5 (3.6)	22.5 (4.0)	21.7 (4.0)
Maximum Tanner stage, n (%)				
1	0	6 (2)	0	6 (2)
2	3 (6)	2 (1)	1 (2)	6 (2)
3	11 (23)	32 (11)	2 (4)	45 (12)
4	21 (45)	112 (42)	26 (52)	169 (44)
5	12 (26)	128 (44)	21 (42)	161 (42)
Race, n (%)				
White	25 (52)	218 (75)	42 (84)	285 (73)
Black or African American	4 (8)	39 (13)	8 (16)	51 (13)
Asian	19 (40)	31 (11)	0	50 (13)
Mixed	0	2 (1)	0	2 (1)
American Indian/Native Alaskan	0	1 (<1)	0	1 (<1)
Axis diagnosis, n (%)				
Schizophrenia	48 (100)	288 (99)	50 (100)	386 (99)
Schizophreniform disorder	0	4 (1)	0	4 (1)
Diagnosis, n (%)				
Paranoid	33 (69)	195 (67)	35 (70)	263 (67)
Undifferentiated	11 (23)	55 (19)	13 (26)	79 (20)
Disorganized	3 (6)	26 (9)	2 (4)	31 (8)
Residual	1 (2)	6 (2)	0	7 (2)
Catatonic	0	6 (2)	0	6 (2)
Schizophreniform disorder	0	4 (1)	0	4 (1)
Age at diagnosis, mean (SD), y	14.8 (1.6)	14.9 (2.2)	15.1 (1.9)	14.9 (2.1)
Age at first psychotic symptoms, mean (SD), y	12.6 (3.1)	13.4 (2.9)	13.6 (2.5)	13.3 (2.9)
Age at start of antipsychotic treatment, mean (SD), y	13.9 (2.6)	14.6 (2.3)	14.8 (1.9)	14.5 (2.3)
Time since onset of first psychotic symptoms, mean (SD), y	2.8 (2.4)	2.1 (2.3)	1.9 (2.0)	2.1 (2.3)

The median mode dose during open-label treatment was 3.8 mg/day (4.0 mg/day, 3.5 mg/day, and 4.0 mg/day in the PBO/RIS, RIS/RIS, and direct-enroll subjects, respectively). For subjects enrolled for 12 months, the median mode dose was 3.0 mg/day. The median duration of exposure was 176 days among subjects enrolled for 6 months, and 336 days for those enrolled for 12 months.

### Efficacy: 6-month data

**Figure 2:**
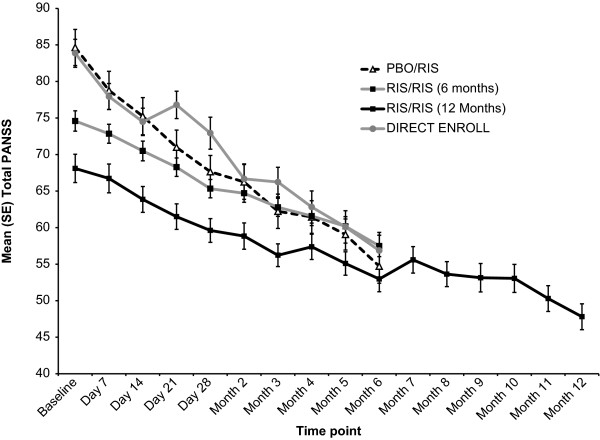
**Positive and Negative Syndrome Scale (PANSS) total score over time (observed cases).** Data are presented as mean (SD). Data are presented for all subjects to the 6- or 12-month end point. The RIS/RIS 6- and 12-month groups are mutually exclusive

From open-label baseline, mean PANSS total scores improved in all three groups (Table 2). Analysis of mean change over time in PANSS total scores (Figure 2) for observed cases indicated that the magnitude of change was most pronounced within the first month of treatment, with maintained improvement over time. Mean score changes from open-label baseline to the 6-month end point indicated improvements in all five PANSS factor scores (Table 2). A clinical response (defined as ≥20% reduction from open-label baseline in PANSS total score) at the 6-month end point was achieved by 61.8% of subjects overall (84.8% of the PBO/RIS, 56.4% of the RIS/RIS, and 72.0% in the direct-enroll groups).

**Table 2:** **Change in PANSS and CGAS from baseline to 6-month end point**^**a**^

	**PBO/RIS**	**RIS/RIS**	**Direct-enroll**	**All subjects**
**PANSS**	**(n = 46)**	**(n = 289)**	**(n = 50)**	**(N = 385)**
**Total**				
Baseline, mean (SD)	84.7 (16.8)	72.1 (19.4)	83.9 (13.5)	75.1 (19.2)
Change, mean (SD)	‐25.7 (18.2)	‐10.5 (17.7)	‐19.9 (16.2)	‐13.6 (18.4)
p value	<-0.001	<-0.001	<-0.001	<-0.001
**Positive symptoms**				
Baseline, mean (SD)	23.6 (6.0)	19.1 (5.7)	23.1 (4.7)	20.1 (5.9)
Change, mean (SD)	-8.7 (6.5)	-3.2 (5.4)	-6.2 (5.2)	-4.2 (5.8)
p value	<-0.001	<-0.001	<-0.001	<-0.001
**Negative symptoms**				
Baseline, mean (SD)	21.5 (5.4)	19.0 (6.5)	21.2 (4.4)	19.6 (6.2)
Change, mean (SD)	-5.7 (5.4)	-2.9 (5.6)	-4.6 (4.1)	-3.4 (5.5)
p value	<-0.001	<-0.001	<-0.001	<-0.001
**Anxiety/depression**				
Baseline, mean (SD)	9.7 (3.3)	8.4 (3.1)	10.1 (3.0)	8.8 (3.2)
Change, mean (SD)	-3.3 (3.2)	-1.2 (3.5)	-2.3 (3.1)	-1.6 (3.5)
p value	< -0.001	< -0.001	< -0.001	< -0.001
**Disorganized thoughts**				
Baseline, mean (SD)	19.4 (4.3)	17.3 (5.7)	19.8 (4.1)	17.9 (5.4)
Change, mean (SD)	-5.0 (4.1)	-2.4 (4.3)	-4.5 (4.3)	-3.0 (4.4)
p value	<-0.001	<-0.001	<-0.001	<-0.001
**Uncontrolled hostility/excitement**				
Baseline, mean (SD)	10.5 (3.6)	8.4 (3.3)	9.6 (3.1)	8.8 (3.4)
Change, mean (SD)	-3.1 (4.0)	-1.0 (3.5)	-2.3 (3.2)	-1.4 (3.6)
p value	<0.001	<0.001	<0.001	<0.001
**CGAS**	(n = 45)	(n = 174)	(n = 41)	(n = 260)
Baseline, mean (SD)	51.8 (16.7)	55.9 (15.5)	51.1 (15.4)	54.4 (15.8)
Change, mean (SD)	16.3 (13.7)	7.5 (14.3)	14.4 (12.2)	10.1 (14.3)
p value	<-0.001	<-0.001	<-0.001	<-0.001

CGAS, Children’s Global Assessment Scale; PANSS, positive and negative syndrome score; PBO, placebo; RIS, risperidone; SD, standard deviation.

^a^Intent-to-treat population, last observation carried forward.

Note: p values are from a within-group test of change from baseline, based on two-sided paired *t* test.

**Figure 3:**
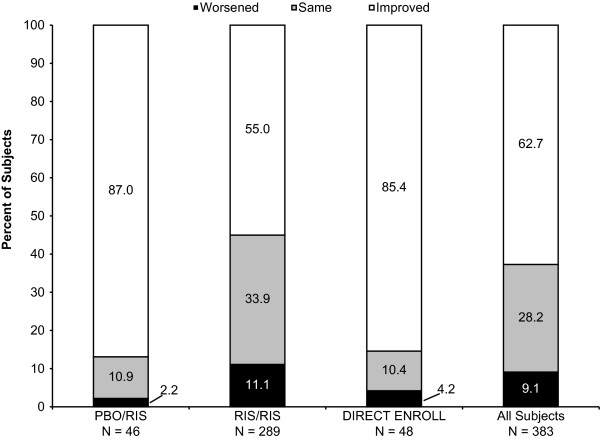
**Percentage of subjects with improved, the same, or worsened clinical status at the 6-month end point, by Clinical Global Impressions of Severity ratings.** Intent-to-treat population, last observation carried forward

Of the total population, 62.7% of subjects were rated as having reduced overall illness severity on the Clinical Global Impressions for Severity at the 6-month end point compared with open-label baseline (Figure 3). Change in CGAS scores at the 6-month end point also showed that all groups experienced functional improvement relative to open-label baseline (Table 2).

As expected, mean symptom and global illness severity scores at the open-label baseline for the efficacy measures were lower for the RIS/RIS group because of significant improvements during double-blind treatment [8,9]. Nevertheless, the majority of RIS/RIS subjects experienced further symptom and functional improvement with continued treatment.

### Efficacy: 12-month data

Subjects enrolled for 12 months demonstrated continued efficacy, as determined by reductions in PANSS total (Figure 2) and PANSS factor scores, as well as improvement in global clinical status and overall functioning.

### Safety

#### Adverse events

All subjects were included in the safety analyses, regardless of whether they were treated for 6 or 12 months. The overall incidence of treatment-emergent AEs was similar in PBO/RIS, RIS/RIS, and direct-enroll groups; most were mild to moderate in severity. The most common were somnolence, headache, weight increase, hypertonia, insomnia, psychosis, and tremor (Table 3).

**Table 3:** **Incidence of treatment-emergent adverse events**^**a**^

**AEs, n (%)**	**PBO/RIS**	**RIS/RIS**	**Direct-enroll**	**All subjects**
	**(n = 48)**	**(n = 292)**	**(n = 50)**	**(N = 390)**
**Treatment-emergent AEs**				
Total	42 (88)	247 (85)	46 (92)	335 (86)
**Most common AEs (≥ 10% in any group)**				
Somnolence	16 (33)	72 (25)	18 (36)	106 (27)
Headache	6 (13)	49 (17)	16 (32)	71 (18)
Weight increase	7 (15)	39 (13)	14 (28)	60 (15)
Hypertonia	7 (15)	37 (13)	8 (16)	52 (13)
Insomnia	7 (15)	35 (12)	4 (8)	46 (12)
Psychosis	3 (6)	31 (11)	7 (14)	41 (11)
Tremor	6 (13)	32 (11)	4 (8)	42 (11)
Rhinitis	4 (8)	29 (10)	1 (2)	34 (9)
Extrapyramidal disorder	5 (10)	23 (8)	2 (4)	30 (8)
Depression	5 (10)	17 (6)	5 (10)	27 (7)
Agitation	5 (10)	19 (7)	2 (4)	26 (7)
Fatigue	1 (2)	17 (6)	8 (16)	26 (7)
Tachycardia	9 (19)	16 (5)	1 (2)	26 (7)
Vomiting	6 (13)	19 (7)	1 (2)	26 (7)
Abdominal pain	1 (2)	10 (3)	9 (18)	20 (5)
**Serious**				
Total	4 (8)	46 (16)	14 (28)	64 (16)
Psychosis	1 (2)	26 (9)	8 (16)	35 (9)
Suicide attempt	1 (2)	14 (5)	3 (6)	18 (5)
Aggressive reaction	0	5 (2)	1 (2)	6 (2)
Injury	0	3 (1)	2 (4)	5 (1)
Depression	0	4 (1)	0	4 (1)
Anxiety	0	2 (1)	1 (2)	3 (1)
Paranoid reaction	0	1 (<-1)	2 (4)	3 (1)
Emotional lability	0	1 (<-1)	1 (2)	2 (1)
Inflicted injury	0	2 (1)	0	2 (1)
Agitation	0	0	1 (2)	1 (<-1)
Convulsions	0	1 (<-1)	0	1 (<-1)
Diabetes mellitus	0	1 (<-1)	0	1 (<-1)
Delusion	0	0	1 (2)	1 (<-1)
Depression psychotic	0	0	1 (2)	1 (<-1)
Drug abuse	0	0	1 (2)	1 (<-1)
Gastrointestinal hemorrhage	1 (2)	0	0	1 (<-1)
Laboratory values abnormal	0	1 (<-1)	0	1 (<-1)
Medication error	0	0	1 (2)	1 (<-1)
Metastases not otherwise specified	1 (2)	0	0	1 (<-1)
Pharyngitis	0	1 (<-1)	0	1 (<-1)
Sarcoma	1 (2)	0	0	1 (<-1)
Schizophrenic reaction	0	1 (<-1)	0	1 (<-1)
Vomiting	1 (2)	0	0	1 (<-1)

AE, adverse event.

^a^Incidence is based on the number of subjects experiencing at least one AE, not the number of AEs.

Serious treatment-emergent AEs occurred in 16% of the overall population (Table 3).

Nineteen subjects had suicide-related AEs; these included attempted suicide (n = 9) or suicidal ideation, thoughts, or tendencies without an actual attempt (n = 10). Among this group of subjects, 2 (4%) were in the PBO/RIS group, 14 (5%) in the RIS/RIS group, and 3 (6%) in the direct-enroll group. All except one of these AEs were considered to be serious by the principal investigator. Of the nine actual suicide attempts, one resulted in death; the details of this event follow.

The subject was a 17-year-old white female, diagnosed with paranoid schizophrenia (baseline PANSS total score = 96) and no other relevant medical history, including no previous documented suicidal behavior. The subject was treated with risperidone (RIS/RIS group) and her dose reached 4 mg/day by study day 3. Her dose was increased over the next 2 weeks to 6 mg/day and then decreased over the next month to 4 mg/day. Concomitant therapies included zopiclone. She had suicidal thoughts (verbatim, “suicidal tendencies”) on study day 5, which resolved by study day 24; she returned home on study day 31. She committed suicide on study day 32, reportedly by jumping from a bridge. Attempts to resuscitate her in the emergency room were not successful. The suicide was assessed by the investigator as a severe event unrelated to study drug. No further follow-up information was available, despite attempts to contact the family.

The most common AEs leading to discontinuation (in 9% of subjects overall) were in the psychiatric disorder class, with only a few AEs reported in more than one subject: psychosis (16 subjects), suicide attempt (nine subjects), aggressive reaction (three subjects), and agitation (two subjects). Increased alanine aminotransferase and EPS each led to discontinuation in two subjects; all other AEs that led to discontinuation were reported in one subject each.

#### Extrapyramidal symptoms

At least one EPS-related AE occurred in 121 (31%) subjects (19 [40%] in the PBO/RIS group, 86 [29%] in the RIS/RIS group, and 16 [32%] in the direct-enroll group). Most common were hypertonia (13%), tremor (11%), extrapyramidal disorder (8%), and hyperkinesia (8%). Dyskinesia occurred in 13 (3%) subjects. Tardive dyskinesia was not reported for any subject. None of the EPS-related AEs was considered serious. Three subjects (all in the RIS/RIS group) discontinued due to EPS-related AEs (two for extrapyramidal disorder, one for dystonia). There were 125 (32%) subjects (16 [33%] in the PBO/RIS group, 85 [29%] in the RIS/RIS group, and 24 [48%] in the direct-enroll group) who used anti-EPS medications during the study. The most frequently used (≥5% of subjects) anti-EPS medications were trihexyphenidyl (11%), benzatropine (7%), biperiden (7%), and diphenhydramine (5%).

Evaluation by the AIMS, BARS, and SAS indicated a low EPS severity at open-label baseline, and no clinically meaningful changes from baseline to the 6-month end point (Table 4). These scales were not in the study protocol before amendment 3, therefore month-12 data were not available.

**Table 4:** **Change in safety parameters from baseline to 6-month end point**^**a**^

**Parameter**	**PBO/RIS**	**RIS/RIS**	**Direct‐enroll**	**All subjects**
	**(n = 48)**	**(n = 292)**	**(n = 50)**	**(N = 390)**
**SAS total score, mean (SD)**	(n = 46)	(n = 180)	(n = 48)	(n = 274)
Baseline	0.03 (0.12)	0.11 (0.24)	0.17 (0.28)	0.11 (0.23)
Change	-0.01 (0.19)	-0.01 (0.28)	-0.05 (0.26)	-0.02 (0.27)
**BARS global rating score, n (%)**				
Baseline	(n = 47)	(n = 180)	(n = 48)	(n = 275)
Absent or questionable	46 (98)	173 (96)	48 (100)	267 (97)
Mild to moderate akathisia	1 (2)	6 (3)	0	7 (3)
Marked to severe akathisia	0	1 (1)	0	1 (<1)
Month 6 end point	(n = 47)	(n = 181)	(n = 50)	(n = 278)
Absent or questionable	47 (100)	176 (97)	50 (100)	267 (98)
Mild to moderate akathisia	0	5 (3)	0	5 (2)
Marked to severe akathisia	0	0	0	0
**AIMS, mean (SD)**	(n = 46)	(n = 180)	(n = 50)	(n = 276)
Baseline	0.4 (1.3)	0.6 (1.8)	0.9 (2.2)	0.6 (1.8)
Change	-0.4 (1.3)	-0.2 (1.4)	-0.3 (2.2)	-0.3 (1.5)
**Prolactin, male subjects, mean (SD), ng/mL**	(n = 28)	(n = 159)	(n = 28)	(n = 215)
Baseline	18.6 (24.7)	40.4 (26.3)	55.8 (25.3)	39.6 (27.6)
Change	29.1 (32.6)	3.7 (28.5)	-6.2 (22.2)	5.7 (29.8)
**Prolactin, female subjects, mean (SD), ng/mL**	(n = 13)	(n = 104)	(n = 18)	(n = 135)
Baseline	14.3 (6.7)	73.6 (43.0)	100.3 (62.3)	71.5 (48.5)
Change	83.4 (44.7)	11.5 (43.3)	-14.0 (49.3)	15.0 (50.1)
**Glucose, mean (SD), mmol/L**	(n = 46)	(n = 267)	(n = 46)	(n = 359)
Baseline	5.1 (0.5)	5.1 (0.7)	5.3 (0.5)	5.2 (0.6)
Change	0.2 (0.8)	0.1 (1.5)	0.0 (0.9)	0.1 (1.4)
**HDL, mean (SD), mmol/L**	(n = 22)	(n = 215)	(n = 46)	(n = 283)
Baseline	1.0 (0.2)	1.3 (0.3)	1.4 (0.4)	1.3 (0.4)
Change	-0.04 (0.2)	-0.05 (0.2)	-0.03 (0.2)	-0.04 (0.2)
**LDL, mean (SD), mmol/L**	(n = 22)	(n = 214)	(n = 46)	(n = 282)
Baseline	2.2 (0.7)	2.4 (0.7)	2.3 (0.5)	2.4 (0.7)
Change	-0.04 (0.4)	0.01 (0.6)	0.04 (0.5)	0.01 (0.5)
**Total cholesterol, mean, (SD), mmol/L**	(n = 46)	(n = 271)	(n = 47)	(n = 364)
Baseline	4.09 (1.07)	4.19 (0.77)	4.20 (0.71)	4.18 (0.81)
Change	0.02 (0.59)	-0.04 (0.73)	-0.03 (0.61)	-0.04 (0.69)
**Triglycerides, mean (SD), mmol/L**	(n = 46)	(n = 271)	(n = 47)	(n = 364)
Baseline	1.19 (0.71)	1.13 (0.58)	1.21 (0.65)	1.15 (0.61)
Change	0.07 (0.58)	-0.00 (0.64)	-0.09 (0.72)	-0.01 (0.64)
**Leptin, mean (SD), μg/L**	(n = 21)	(n = 217)	(n = 47)	(n = 285)
Baseline	11.4 (11.1)	13.1 (14.2)	12.3 (14.3)	12.9 (14.0)
Change	6.5 (12.8)	2.0 (9.3)	2.6 (7.2)	2.4 (9.3)
**Height, mean (SD), cm**	(n = 44)	(n = 278)	(n = 49)	(n = 371)
Baseline	163.9 (10.2)	167.3 (11.4)	173.0 (9.8)	167.6 (11.3)
Change	0.9 (1.3)	1.0 (1.6)	1.1 (1.5)	1.0 (1.6)
**Weight, mean (SD), kg**	(n = 44)	(n = 278)	(n = 49)	(n = 371)
Baseline	60.2 (21.3)	62.6 (13.7)	67.6 (14.3)	63.0 (14.9)
Change	4.3 (5.5)	3.9 (5.3)	4.5 (5.8)	4.0 (5.4)
**BMI, mean (SD), kg/mÂ²**	(n = 44)	(n = 278)	(n = 49)	(n = 371)
Baseline	22.0 (5.9)	22.2 (3.7)	22.5 (3.9)	22.2 (4.0)
Change	1.4 (1.9)	1.1 (1.9)	1.2 (1.7)	1.2 (1.8)

^a^Intent-to-treat population, last observation carried forward.

AIMS, Abnormal Involuntary Movement Scale; BARS, Barnes Akathisia Rating Scale; BMI, body mass index; HDL, high-density lipoprotein; LDL, low-density lipoprotein; PBO, placebo; RIS, risperidone; SAS, Simpson-Angus Scale; SD, standard deviation.

#### Somnolence/fatigue

Somnolence was noted in 27% of subjects overall (33% in the PBO/RIS group, 25% in the RIS/RIS group, and 36% in the direct-enroll group). Fatigue occurred in 7% overall (2% in the PBO/RIS group, 6% in the RIS/RIS group, and 16% in the direct-enroll group). Apart from one subject with somnolence and one with fatigue, all cases were rated as mild to moderate. No subjects discontinued because of either somnolence or fatigue. Most of these events began in the first 2 weeks of treatment and lasted a median of 14 and 11 days, for somnolence and fatigue, respectively. Most subjects (91.9%) recovered from somnolence/fatigue during the study.

#### Prolactin

Potentially prolactin-related AEs (PPAEs) were reported by 36 (9%) subjects. Of these, 18 had a report of hyperprolactinemia based on abnormal laboratory values but had no clinical symptom related to prolactin. Another 18 had a report of a clinical symptom potentially related to prolactin (five of whom also had a report of hyperprolactinemia). These AEs included gynecomastia (five male subjects), nonpuerperal lactation (nine female subjects), amenorrhea (three female subjects), breast pain (two female subjects), and decreased libido (one male subject) [some subjects had multiple events]. PPAEs were the cause of discontinuation for two subjects, one for hyperprolactinemia and one for nonpuerperal lactation. Dose reductions were implemented for seven subjects, and one subject received concomitant therapy for amenorrhea. PPAEs resolved in 15/43 (34.9%) patients during the study, and all were mild or moderate in severity. Twenty-eight of 43 PPAEs had not resolved by the end of the study; these included asymptomatic hyperprolactinemia (18 subjects), gynecomastia (three subjects), nonpuerperal lactation (four subjects), and amenorrhea (three subjects).

Mean prolactin levels at open-label baseline were higher in risperidone-naive male subjects than in female subjects, but changes during the study were more notable in females (Table 4). In all three groups, mean prolactin peaked at month 1; small mean decreases were observed thereafter. This is consistent with previous risperidone studies in other populations.

#### Metabolic effects

Two subjects reported glucose-related AEs. The first subject had mild abnormal glucose tolerance and serious diabetes mellitus reported as AEs. The subject had a family history of diabetes and was overweight; body weight (BMI) was 90.4 kg (36.4 kg/m^2^) at baseline and 99.0 kg (39.9 kg/m^2^) at the 6-month end point. The subject had elevated glucose levels at open-label baseline and throughout the study. The subject was withdrawn from the study on day 118. Twenty-one days after the last dose of risperidone, the glucose level was below the baseline level but still above the normal range. The second subject completed 168 days of open-label treatment. Mild abnormal glucose tolerance was reported as an AE the day after receiving the last dose of study drug (day 169); no follow-up laboratory data were available after study completion.

Mean fasting glucose levels were similar in all three groups at open-label baseline. A small increase was observed at the 6-month end point (Table 4). At the 12-month time point, the mean (SD) change from baseline in glucose levels was -0.1 mmol/L (0.1) for 47 subjects who enrolled before amendment 3.

Weight increase was reported as a treatment-emergent AE for 60 (15%) subjects. The incidence was 15% in the PBO/RIS group, 13% in the RIS/RIS group, and 28% in the direct-enroll group. In all but three subjects, weight increase was rated as mild or moderate in severity. One subject in the PBO/RIS group discontinued treatment due to weight increase of moderate severity (19.6 kg at day 134 of the study).

**Figure 4:**
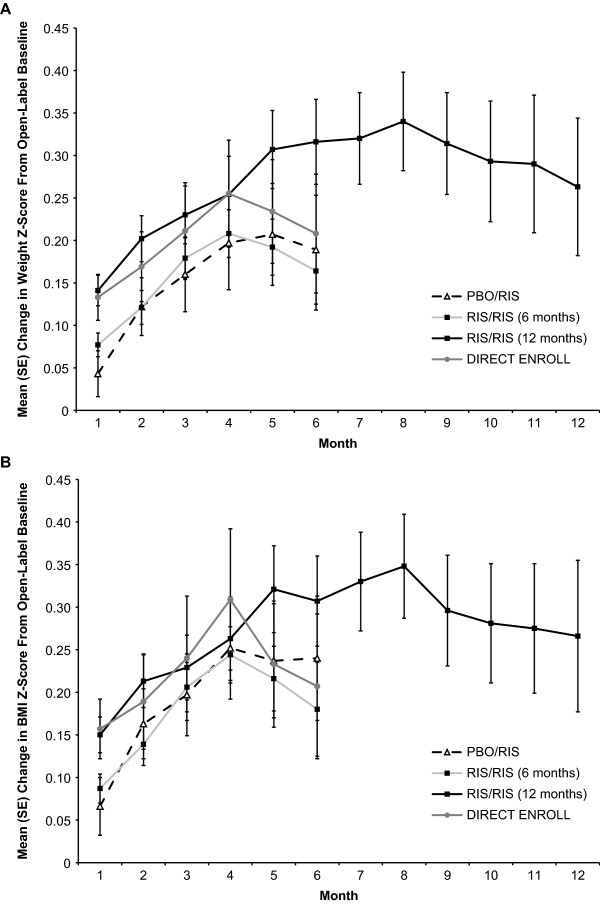
**Mean change in*z*scores for body weight (A) and body mass index (B) over the course of the study for the PBO/RIS group, RIS/RIS group, and all subjects.** Data are presented for all subjects to the 6- or 12-month end point. The RIS/RIS 6- and 12-month groups are mutually exclusive

Mean body weight, height, and BMI increased similarly in all groups from open-label baseline to 6-month end point (Table 4). Mean changes in *z* scores for weight and BMI over time are shown in Figure 4. Increases in weight and BMI appeared to be greatest in the first several months of treatment, and appeared to plateau after 3 to 4 months.

#### Other safety parameters

No clinically meaningful changes were observed in mean high-density lipoprotein, low-density lipoprotein, total cholesterol, or triglyceride levels between groups (Table 4).

Leptin, which has been demonstrated to correlate with weight gain in some studies [26-29], showed a mean increase from baseline to end point in the earlier 8-week controlled study and also in this 6-month open-label extension study (Table 4). As expected, increases in leptin were positively correlated with increases in body weight. At the 12-month time point (n = 47), the mean (SD) change from baseline in leptin levels was 2.7 (2.2) μg/L.

Throughout this 6-month-or-longer study, Tanner staging assessments reflected a shift from open-label baseline consistent with expected developmental maturation in both male and female subjects.

No clinically meaningful changes were detected in the mean electrocardiogram parameters or corrected QT intervals. The mean (SD) change from baseline to the month 6 end point in QTcF (Fridericia correction) was 0.0 (17.7) for the PBO/RIS group, 0.3 (17.2) for the RIS/RIS group and 3.9 (11.1) in the direct enroll group. There was one female subject in the RIS/RIS group who went from normal QTcF (420 ms) to prolonged (460 ms) during the study.

#### Onset of AEs

The proportion of subjects with any treatment-emergent AE with onset during the first month of open-label treatment was greater among subjects previously treated with placebo (81%) and in directly enrolled subjects (80%), compared with subjects previously treated with risperidone (in the double-blind study) (67%). Certain AEs (including somnolence, insomnia, and tremor) were reported more often during the first month of open-label treatment, compared with subsequent treatment periods (Table 5). The proportion of subjects reporting somnolence during the first month of treatment was greater among subjects previously treated with placebo and directly enrolled subjects (~30%), compared with subjects previously treated with risperidone (19%).

**Table 5:** Incidence of treatment-emergent AEs by time period

**Month of onset of AE**	**0-1**	**>1-2**	**>2-3**	**>3-6**	**>6-9**	**>9-12**	**>12**
**Subjects at each time point, n**							
PBO/RIS	48	45	44	43	22	0	0
RIS/RIS	292	266	254	242	136	67	42
Direct-enroll	50	44	41	40	19	0	0
**Any AE, n (%)**							
PBO/RIS	39 (81)	11 (24)	9 (20)	21 (49)	3 (14)	–	–
RIS/RIS	197 (67)	82 (31)	80 (31)	117 (48)	51 (38)	33 (49)	8 (19)
Direct-enroll	40 (80)	21 (48)	18 (44)	23 (58)	3 (16)	–	–
**Somnolence, n (%)**							
PBO/RIS	15 (31)	1 (2)	2 (5)	1 (2)	0	–	–
RIS/RIS	55 (19)	4 (2)	8 (3)	9 (4)	2 (1)	3 (4)	0
Direct-enroll	16 (32)	4 (9)	2 (5)	2 (5)	0	–	–
**Tremor, n (%)**							
PBO/RIS	6 (13)	0	0	0	0	–	–
RIS/RIS	23 (8)	2 (1)	3 (1)	5 (2)	2 (1)	0	0
Direct-enroll	3 (6)	1 (2)	0	0	0	–	–
**Insomnia, n (%)**							
PBO/RIS	5 (10)	1 (2)	0	1 (2)	0	–	–
RIS/RIS	24 (8)	4 (2)	5 (2)	8 (3)	4 (3)	2 (3)	1 (2)
Direct-enroll	3 (6)	1 (2)	1 (2)	1 (3)	0	–	–
**Hypertonia, n (%)**							
PBO/RIS	6 (13)	0	0	0	2 (9)	–	–
RIS/RIS	27 (9)	1 (<1)	6 (2)	7 (3)	0	0	0
Direct-enroll	6 (12)	1 (2)	0	2 (5)	0	–	–
**Weight increase, n (%)**							
PBO/RIS	1 (2)	1 (2)	0	5 (12)	0	–	–
RIS/RIS	15 (5)	6 (2)	5 (2)	10 (4)	4 (3)	2 (3)	0
Direct-enroll	11 (22)	2 (5)	1 (2)	3 (8)	0	–	–

PBO, placebo; RIS, risperidone.

## Discussion

This open-label study examined the efficacy and safety of continued risperidone treatment in adolescent subjects with schizophrenia. Of the 390 subjects who entered this study, 292 had been treated with risperidone in one of two previous randomized double-blind studies. Important amendments to the study (most notably relating to duration in the study), as well as the multiple sources of enrollment, complicate its interpretation and possibly make the data less generalizable.

Generally, at doses of 2 to 6 mg/day, risperidone was associated with improvements in symptoms and functioning. Improvement was greater during the first month and was generally sustained throughout the treatment period.

As anticipated, PBO/RIS and direct-enroll subjects appeared to have a greater degree of symptomatic improvement compared with subjects who had received previous double-blind risperidone treatment (RIS/RIS subjects). RIS/RIS subjects had already experienced significant improvements by the time they entered the open-label study and continued to show improvement throughout the open-label study. Results were consistent across different measures of symptoms and functioning. The majority of subjects enrolled showed symptomatic improvement, then stability.

Subjects who enrolled for 12 months demonstrated continued efficacy, as determined by reductions in PANSS total and factor scores as well as by improvement in global clinical status and overall functioning. To our knowledge, this was the first documentation of the potential benefit of longer-term treatment in adolescents with schizophrenia.

In this study, the dose was increased to the maximum tolerated dose within the allowable range, mirroring common clinical practice. The dose range in this study, 2 to 6 mg/day, is comparable to that utilized in adult populations [30-34], and the median dose in this study (3.8 mg/day) is similar to the recommended dose of 3 mg/day based on the short-term controlled studies [8,9].

Risperidone treatment was generally well tolerated, and no new safety concerns were identified. Evidence of tolerability included stability of doses at clinically relevant levels and minimal need for dose reduction. There was also an overall high rate of study completion (>65%) and a low rate of AE-related discontinuation (9%). The majority of AEs were mild or moderate in severity. The qualitative nature of the AEs, both reported and measured, was similar to those noted with risperidone in adult subjects and in other pediatric populations. Findings for AEs of particular concern were consistent with previous findings in long-term studies of risperidone in children or adolescents with conduct disorders or autism spectrum disorders [35-39]. Most subjects had been treated with risperidone in their previous study and would be expected to have already established tolerability upon entering this open-label study,

Somnolence was limited mainly to the start of open-label treatment. No cases of tardive dyskinesia were reported, and the severity of EPS, as measured by both AE reporting and clinical rating scales, was low throughout the study. Prolactin findings were similar to those reported in other studies across a variety of indications and age groups. Small changes in glucose and lipid levels were measured. Change in weight and BMI *z* scores exceeded expected gains during the first few months of treatment, and then leveled-off over time. Sexual maturation appeared not to be affected by risperidone treatment.

### Study limitations

The open-label study design used here is known to be associated with potential bias when interpreting study results. Subjects were titrated to the maximum tolerated dose, which may have resulted in higher rates for some AEs. Previous treatment with risperidone in some subjects may have resulted in a lower frequency of certain AEs reported with subsequent exposure. Previously exposed subjects may have already experienced such events early on in their treatment (before the reporting period of this trial) or potentially may have discontinued. It is also possible that longer-term treatment (i.e., longer than 12 months) could be associated with additional AEs (e.g., metabolic parameters, growth or sexual maturation) that may not have been observed in the study.

## Conclusions

Maintenance of treatment requires careful consideration of benefit and risk. Current recommendations for long-term antipsychotic treatment in adolescents with schizophrenia have relied on limited available research, the adult literature and clinical experience [40].

This study provides longer-term safety and efficacy data for antipsychotic treatment in a population of severely impaired adolescents with schizophrenia. Safety and tolerability findings appeared consistent with findings in adult studies, as well as in other pediatric populations including adolescents with disruptive behavior disorders. Although these data need to be interpreted with caution, these findings suggest that risperidone treatment of 6 to 12 months is generally well tolerated and may provide sustained improvement in symptoms as well as global functioning in adolescents with schizophrenia.

## Abbreviations

AE: adverse event; AIMS: Abnormal Involuntary Movement Scale; BARS: Barnes Akathisia Rating Scale; CGAS: Children’s Global Assessment Scale; *DSM-IV*: *Diagnostic and Statistical Manual of Mental Disorders*, 4th edition; EPS: extrapyramidal symptoms; PANSS: Positive and Negative Syndrome Scale; PBO: placebo; RIS: risperidone; SAS: Simpson-Angus Scale; SD: standard deviation.

## Competing interests

GP, KK, and MH are employees of Janssen Research & Development, LLC and are Johnson & Johnson stockholders. SK was an employee of Janssen Research & Development, LLC at the time of this analysis.

## Authors’ contributions

MH, and KK contributed to the conception and design and acquisition of data, GP and SK additionally contributed to the analysis and interpretation of data, and drafting of the manuscript and its critical revision for important intellectual content. All authors read and approved the final manuscript.
